# COVID-19 Related Retinal Vascular Occlusion: A Systematic Review

**DOI:** 10.3390/jcm14041183

**Published:** 2025-02-11

**Authors:** Argyrios Tzamalis, Maria Foti, Maria Georgiadou, Nikolaos Tsaftaridis, Nikolaos Ziakas

**Affiliations:** 2nd Department of Ophthalmology, Aristotle University of Thessaloniki, Papageorgiou General Hospital, 564 29 Thessaloniki, Greece; mariafoti@auth.gr (M.F.); mtgeorgiad@gmail.com (M.G.); tsaftaridis1@gmail.com (N.T.); nikolasziakas@gmail.com (N.Z.)

**Keywords:** COVID-19, SARS-CoV-2, vaccination, retinal vascular occlusion, vein occlusion, artery occlusion

## Abstract

**Background/Objectives**: To provide insight into populations at higher risk of COVID-19-related retinal vascular occlusion, we identified the baseline characteristics of COVID-19 patients and vaccine recipients who developed this condition by conducting a systematic review to summarize the findings and evaluate the current knowledge on this subject. **Methods**: An electronic search on the PubMed and Scopus databases was performed for relevant case reports or series regarding retinal vascular occlusion in patients with past or present COVID-19 infection or SARS-CoV-2 immunization. This study was conducted using a pre-determined protocol following the Preferred Reporting Items for Systematic Reviews and Meta-Analyses (PRISMA) guidelines. **Results**: A total of 34 studies were enrolled in this systematic review. A total of 21 patients (14 males, 66.7%) have been diagnosed with COVID-19 related retinal vein occlusion (RVO, mean age = 41.9 ± 10.3 years), and 15 patients (12 males, 80%) have been diagnosed with retinal artery occlusion (RAO, mean age = 56.9 ± 13.2 years). The time to RVO since COVID-19 infection or SARS-CoV-2 immunization ranged from 8 h to 51 days (mean = 12.3 ± 15.7 days), while the time to RAO ranged from 2 to 40 days (mean = 14.9 ± 10.8 days). Fifteen out of the twenty-one patients (71.4%) with RVO had a significant improvement in visual acuity after the resolution of symptoms while eight out of the fifteen patients (53.3%) with RAO did not show improvement. **Conclusions**: COVID-19 seems to play a significant role in the pathogenesis of vascular occlusion, as it is suggested to increase the risk of thromboembolic episodes. However, the pathophysiologic mechanisms have not been fully elucidated, and further studies are expected to shed light on this phenomenon.

## 1. Introduction

Although COVID-19 affects mainly the respiratory system, it has also been associated with coagulopathies and venous or arterial thromboembolism [1,2]. Moreover, SARS-CoV-2 infection is associated with ocular manifestations, such as conjunctivitis (most common), changes in the retinal vasculature, and ocular thromboembolic events [3].

Retinal vascular occlusions are divided into two main categories: Retinal vein occlusions (RVO) and retinal artery occlusions (RAO), the former being much more common than the latter, with a better prognosis. Common risk factors for both types of occlusions are age over 50 years and the presence of cardiovascular risk factors [4]. Most acute artery occlusions are embolic, secondary to internal carotid artery disease. Non-embolic occlusions can occur from vasculitis and infectious causes. On the other side, predisposing factors for retinal vein thrombosis are hypertension, diabetes, arterial nicking (narrowing), and glaucoma [5].

The first case of ocular vascular occlusion (OVO) related to COVID-19 infection was published only some months after the pandemic outbreak, in June 2020 [6], while the first case of OVO related to COVID-19 vaccination was reported in January 2022 [7], and soon additional, similar case reports started emerging [8,9,10,11,12,13,14,15,16,17,18,19,20,21,22,23,24,25,26,27,28,29,30,31,32,33,34,35,36,37,38,39]. Although several cases have already been published on this subject, there is still controversy regarding the true relationship between COVID-19 and retinal vascular occlusion events. Therefore, we have conducted a systematic review attempting to summarize the findings and evaluate the current knowledge on this topic.

The primary objective of this study was to identify the baseline characteristics of COVID-19 patients and vaccine recipients who are more likely to develop retinal vascular occlusion, attempting to provide insight into populations at higher risk. The secondary objective was to thoroughly review these cases’ presenting clinical images, laboratory examinations, management strategies, and outcomes, attempting to evaluate the association between COVID-19 and retinal vascular occlusion.

## 2. Methods

### 2.1. Conduct of Review

This study was conducted in line with a pre-determined protocol agreed upon by all co-authors, following the Preferred Reporting Items for Systematic Reviews and Meta-Analyses (PRISMA) guidelines.

### 2.2. Search Strategy and Study Selection

**Figure 1:**
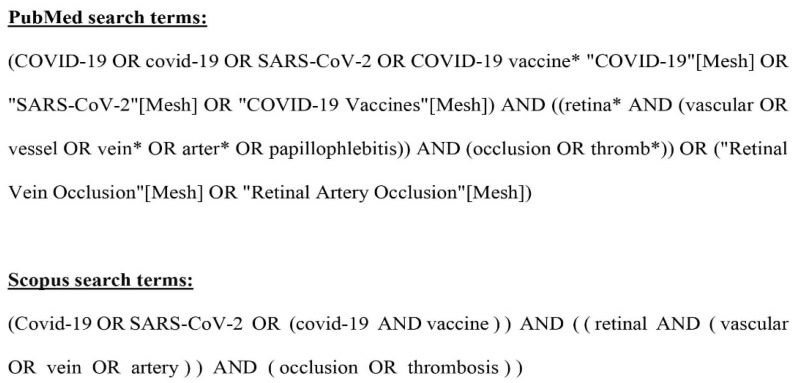
Search terms used for conducting this systematic review.

An electronic search on the PubMed and Scopus databases was performed (end date 3 November 2024) for relevant case reports or case series regarding retinal vascular occlusion in patients with past or present COVID-19 infection or SARS-CoV-2 immunization. The search terms used are thoroughly presented in Figure 1. Time restrictions were applied from 2019 to that date, and no linguistic restrictions were used. Subsequently, all relevant articles were screened for eligibility against the inclusion/exclusion criteria by three independent reviewers (NT, MG, MF). Articles were included if they involved patients with a history of COVID-19 infection or SARS-CoV-2 immunization and if they reported on retinal vascular occlusion in those patients. Articles were excluded if they did not present original data, had incomplete data, or did not meet certain additional eligibility criteria. These criteria encompassed articles not available in English, reporting on booster vaccinations, involving patients under 16 years old, and articles without confirmed COVID-19 infection or vaccination before or at the onset of ocular symptoms. Additional exclusions were applied for articles involving conditions beyond COVID-19 and retinal vascular occlusion, for articles with combined retinal vascular occlusion, and for articles where an additional COVID-19 infection or vaccination occurred after the retinal vascular occlusion, as these could confound the results. Finally, the three independent reviewers systematically reviewed the reference lists of eligible manuscripts (“snowballing”) for potentially eligible articles. The search aimed to find original articles describing retinal vascular occlusion in COVID-19 patients or vaccine recipients. For this reason, review articles and correspondence presenting original data were included. Institutional review board approval was obtained before data extraction. The research methodology complied with the principles of the Declaration of Helsinki. The requirement for informed consent was waived due to the retrospective nature of the study.

### 2.3. Data Extraction

The articles included in the study were reviewed by three independent reviewers. Data were extracted and organized into a pre-piloted, standardized form. Each reviewer independently extracted the data, and any discrepancies identified were resolved through consensus, with input from a senior author (AT). We identified and extracted the following data for analysis from each paper, where available: number of patients, age, weight, BMI, sex, comorbidities, type of ophthalmic vascular occlusion, occlusion symptoms, vaccine type, method of COVID-19 diagnosis, laboratory exams, previous COVID-19 diagnosis status, relevant imaging studies, exam findings, clinical features, management, and outcome.

### 2.4. Statistical Analysis

Statistical analysis was performed with Medcalc statistical software (version 9.3.0.0, Medcalc, Mariakerke, Belgium) and SPSS (v. 22.0 for Windows, SPSS INC, Chicago, IL, USA). Normality was checked using the Kolmogorov–Smirnov test. Since the data were normally distributed for all continuous parameters tested, mostly parametric methods were used. Descriptive statistics were employed to summarize all variables. Categorical variables are represented as frequencies and percentages, while continuous variables are summarized as means and standard deviations (SDs). All relative rates were calculated using the available data for the variables of interest, and all analyses were conducted following the principles outlined in the Cochrane Handbook [40].

## 3. Results

**Figure 2:**
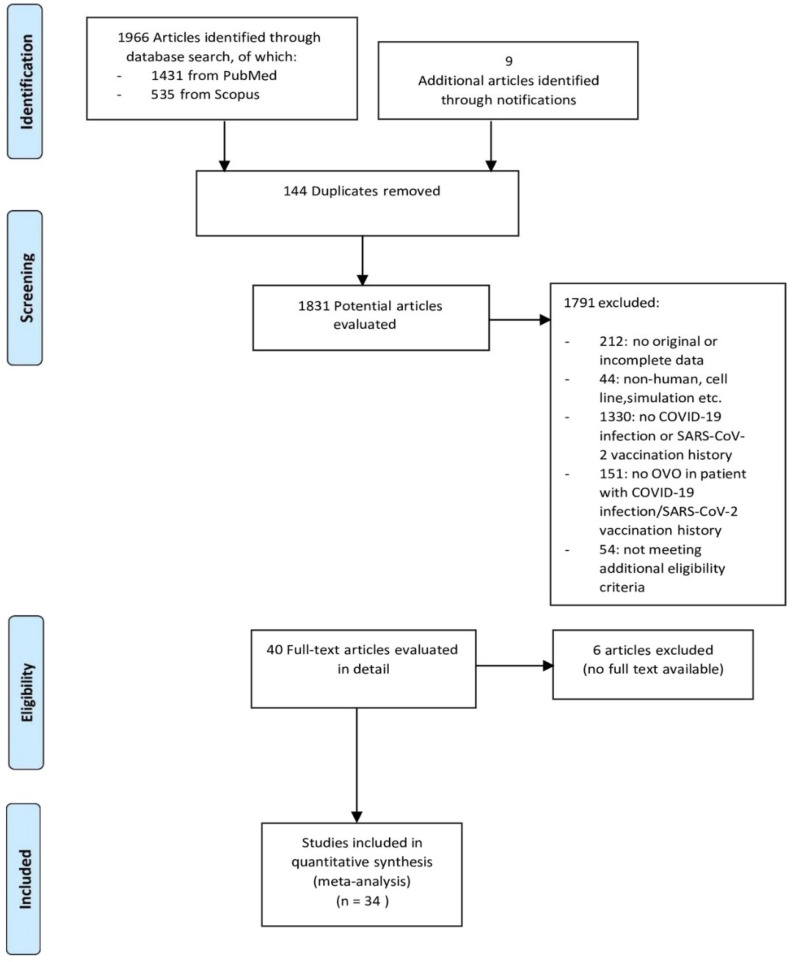
PRISMA Flowchart.

In total, we identified 1975 potentially relevant articles in the initial database search. From these, 1941 were excluded following screening, as they were duplicates or not relevant. A total of 34 studies were finally enrolled in this systematic review (Figure 2). Among these, 32 were reports of single cases and 2 were case series, each one including reports of 2 cases. Thorough demographic data of the patients and details of each study are provided in Table 1 and Table 2.

We further divided retinal vascular occlusion cases into artery (RAO) and vein (RVO) ones. A total of 21 patients (14 males, 66.7%) aged between 17 to 59 years old (mean ± SD = 41.9 ± 10.3 years, Table 1) have been diagnosed with RVO. Among these, 13 cases of retinal vein occlusion (61.9%) occurred after COVID-19 infection, while 8 cases (38.1%) were noted after vaccination. The time to RVO since COVID-19 infection or SARS-CoV-2 immunization ranged from 8 h (1/3 day) to 51 days (mean ± SD = 12.3 ± 15.7 days), with all of the patients complaining about decreased vision or respective visual field defects. The imaging techniques used for the diagnosis of RVO included fundoscopy, optical coherence tomography (OCT), and fluorescein angiography (FA). Nine out of twenty-one patients underwent all three imaging techniques, nine had only fundoscopy and OCT, two patients were examined with fundoscopy and FA, and one patient had only fundoscopic examination available. Twelve out of twenty-one patients were previously healthy, and eight had systemic comorbidities such as obesity, diabetes, hypertension, polycystic ovary syndrome (PCOS), microscopic colitis, chronic kidney disease (CKD), and cancer. One patient had Coats disease as a comorbidity. The laboratory workup showed that six patients had elevated inflammatory markers (6/21, 28.6%) and six patients had elevated coagulation markers (D-dimers, 6/21, 28.6%).

**Figure 3:**
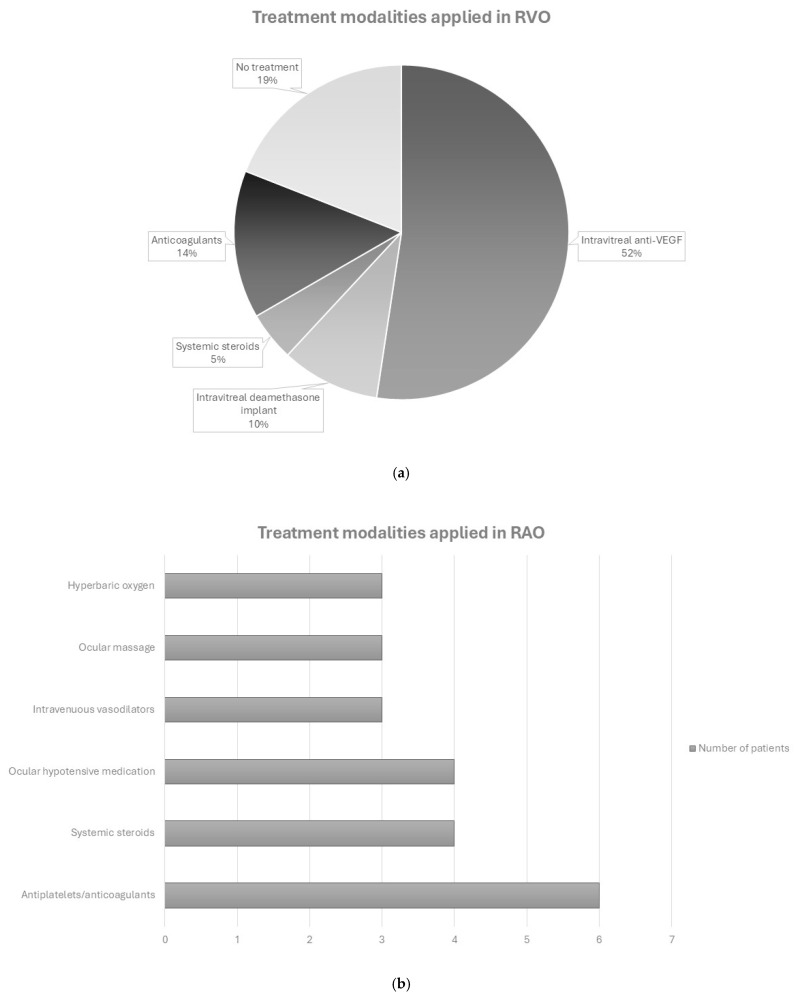
Treatment modalities applied in cases of retinal vein occlusion (RVO, (**a**)), and retinal arterial occlusion (RAO, (**b**)) related to COVID-19 infection or immunization.

Various treatment modalities were applied to these patients (Figure 3a). One patient was treated with oral methylprednisolone along with intravitreal anti-VEGF injections; one with intravitreal anti-VEGF and periocular triamcinolone injections along with focal laser; nine patients received only intravitreal anti-VEGF injections; one patient was treated with low molecular weight heparin (LMWH) along with rivaroxaban; one was treated only with aspirin; another patient received aspirin, bromfenac eye drops, and an intravitreal dexamethasone implant; another patient was treated only with an intravitreal dexamethasone implant; four patients received no treatment; and for two patients there were no available data. Fifteen out of the twenty-one patients (15/21, 71.4%) had a significant improvement in visual acuity after the resolution of symptoms while two patients maintained their optimal visual acuity both at the onset of retinal vein occlusion and at follow-up. There were no available data for the other four cases.

As far as retinal artery occlusions are concerned, a total of 15 patients (12 males, 80%) aged between 37 to 76 years old (mean ± SD = 56.9 ± 13.2 years, Table 2) have been reported with COVID-19 related RAO. Among these, 12 cases of retinal artery occlusion (80%) occurred after COVID-19 infection, while 3 cases (20%) were reported after vaccination. The time to RAO since COVID-19 infection or SARS-CoV-2 immunization ranged from 2 to 40 days (mean ± SD = 14.9 ± 10.8 days), with the majority of patients (12/15, 80%) complaining of painless vision loss. One patient had no ocular symptoms. The imaging techniques used for diagnosis included fundoscopy, OCT, FA, brain and orbit computed tomography angiography (CTA), head magnetic resonance imaging (MRI), and visually evoked potentials. Six out of the fifteen patients were previously healthy and the other nine had comorbidities such as diabetes, hypertension, dyslipidemia, hyperuricemia, coronary artery disease, chronic obstructive pulmonary disease, hypothyroidism, and sickle cell trait. Six out of the fifteen RAO patients (6/15, 40%) had elevated inflammatory markers, and eight (8/15, 53.3%) did not show improvement. For each patient a different intervention was used (Figure 3b): a combination of aspirin and dexamethasone; LMWH; intravenous (IV) steroids with antibiotics and anticoagulants and symptomatic care; topical prednisolone in a patient with concurrent bilateral panuveitis; ocular massage with hypotensive eye drops; ocular massage with hypotensive eye drops and hyperbaric oxygen therapy; ocular massage with hypotensive eye drops, aspirin, and IV vasodilator; IV vasodilator; clopidogrel and hyperbaric oxygen therapy; a combination of prednisolone and hypotensive eye drops and panretinal photocoagulation; and a combination of aspirin, prednisolone, IV vasodilator, retrobulbar anticholinergic injections, anterior chamber puncture, and supplemental oxygen. Three patients received no treatment and for one patient there were no available data.

## 4. Discussion

Retinal vascular occlusions are classified among the most common causes of visual loss that occur in people aged >50 years, usually with additional cardiovascular risk factors, such as hypertension and diabetes [4]. CRAO can be either embolic or non-embolic. More commonly, emboli from the heart due to atrial fibrillation and internal carotid artery atherosclerosis lead to acute obstruction of the arterial retinal blood flow, similar to cerebral infarctions. Thus, acute CRAO can lead to irreversible cell death and a permanent decrease in vision within a few hours [5]. A non-embolic way that carotid artery atherosclerosis can cause CRAO is through a significant stenosis of the vessel diameter (>70%), thus reducing the ocular blood flow. Moreover, it is hypothesized that the platelet aggregation in the atherosclerotic plaque results in the release of serotonin, which is a powerful vasoconstrictor and can produce a transient spasm in the retinal arteries [41].

Other non-atherosclerotic causes of CRAO include systemic diseases, such as vasculitis and giant cell arteritis, and hematological diseases such as sickle cell anemia, leukemia, and lymphoma [5]. Autoimmune mediated and infectious disorders, as well as thrombophilia, are also related to CRAO, which has also been reported following hemodialysis and orbital and eye surgery or head injuries [5,41].

Risk factors for central retinal vein occlusion (CRVO) include hypertension, diabetes, glaucoma, and cardiovascular disease [5]. Although the pathogenesis of CRVO is not completely understood, it is considered that the venous obstruction occurs in the region of or most likely just posterior to the lamina cribrosa. It is considered that when the occlusion of the central retinal vein occurs in the lamina cribrosa, it usually causes a more complete obstruction and, therefore, a worse prognosis, while an occlusion posterior to the lamina cribrosa leads to less severe or non-ischemic obstruction [42]. Increased intraocular pressure in particular can displace the lamina cribrosa and change the shape of the central retinal vein, leading to increased turbulence and endothelial stress [5]. Moreover, the central retinal vein can be compressed by a rigid retinal arterial wall, especially in those with atherosclerosis. This occurs because the central retinal artery and vein share a common adventitial sheath [42]. Other predisposing factors include systemic vascular comorbidities and a prothrombotic state [5]. Interestingly, inflammation, dehydration, and exercise can also lead to CRVO in younger people with congenital anomalies but without any other comorbidity [42].

SARS-CoV-2 uses angiotensin-converting-enzyme-2 (ACE-2) receptors to release its RNA inside the host’s epithelial cells [43,44,45]. After the virus has infected the epithelial cells of the lungs, it can enter the bloodstream and travel throughout various parts of the body, including the heart, brain, gastrointestinal tract, kidney, and liver, thus causing symptomatology from the affected organs, such as cerebral hemorrhage and ischemic stroke. Once the virus infects the epithelial cells, it releases cytokines that lead to localized inflammation, endothelial activation, and tissue damage [45]. Zhou et al. have shown that the ACE-2 receptor is also expressed in many non-vascular retinal cells, such as the photoreceptor outer segments, the inner nuclear layer, the inner plexiform layer, and the retinal ganglion cell layer. Furthermore, they observed that there is increased expression of vascular ACE-2 in the retinas of diabetic retinopathy patients [46].

In the case reports that we examined [6,7,8,9,10,11,12,13,14,15,16,17,18,19,20,21,22,23,24,25,26,27,28,29,30,31,32,33,34,35,36,37,38,39], around half of RAO and one-third of RVO patients had at least one risk factor, and the remaining patients were individuals without any comorbidity or medication use associated with an increased risk of retinal vascular occlusion. We hypothesize that SARS-CoV-2 infection or immunization, through the release of inflammatory cytokines, has resulted in CRAO or CRVO in patients with other cardiovascular risk factors. Moreover, SARS-CoV-2 may have reached the retinal cells of previously healthy patients, thus causing CRVO and CRAO in younger people through the release of inflammatory cytokines, since inflammation and infectious disorders have already been related to CRVO and CRAO, respectively [5,43,45]. However, further research is essential to understand the link between COVID-19 infection or vaccination and retinal vascular occlusion.

Interestingly, the case of a 58-year-old patient diagnosed with BRAO who did not complain of any symptoms has been reported [17]. This suggests that there may be some subclinical cases of retinal obstruction that go undetected. Another possibility could be that some patients may not be detected because more serious types of thrombosis, such as deep vein thrombosis, mask the presentation of retinal vascular obstruction as a primary clinical concern.

Clinical presentation of COVID-19 related retinal vascular occlusions reviewed in this study did not seem to differ significantly from non-COVID respective cases, as most patients demonstrated decreased vision in vein occlusions and painless vision loss in arterial ones. A male predominance was noticed for both RVO (14/21, 66.7%) and RAO cases (12/15, 80%). Regarding patients’ age, patients with COVID-19 related RAO seemed to be older (56.9 ± 13.2 years old) than patients presenting with vein occlusions during or after their COVID-19 infection or SARS-CoV-2 vaccination (41.9 ± 10.3 years old) and this difference was found to be statistically significant (Students *t*-test, *p* = 0.001).

Regarding management, several therapeutic modalities have been applied to patients with COVID-related retinal occlusions. The most common among cases reported in this review were anti-VEGF intravitreal injections in vein occlusions, especially in the presence of macular edema, and antiplatelet/anticoagulant medication in arterial ones (Figure 3a,b). The type of treatment, however, did not seem to play a substantial role in terms of visual acuity improvement. The final visual outcome of vein occlusions was much more favorable than that of arterial occlusions, as expected.

Thrombotic complications such as acute limb ischemia due to COVID-19 usually present around 13 days according to one study by Topcu et al. [47], while Fournier et al. estimated the time to presentation at 11 days, with a range of 5 to 20 days [48]. In the case studies included in this review, arterial events in COVID-19 patients and vaccine recipients were detected 2 to 40 days after infection or vaccination (mean ± SD = 14.9 ± 10.8 days). Fournier et al. also noted that arterial thrombotic events in COVID-19 seem to be associated with typical cardiovascular risk factors, and that in these patients, mortality is higher [48]. This may be indicative of the assumption that COVID-19 may need “fertile ground” to cause arterial occlusions.

Venous thromboembolism is, similar to arterial events, associated with common risk factors, such as male sex, older age, elevated D-dimers, coronary artery disease, and prior myocardial infarction [49]. It is known that complement activation is increased in COVID-19 patients compared to other causes of pneumonia, suggesting a possible pathophysiologic mechanism implicating local inflammatory responses and the endothelium in venous thrombosis [50,51,52]. Retinal vein occlusion could also be attributed to comorbidities of the patients included in this study rather than COVID-19 infection or immunization. For instance, Garduno et al. reported on a 43-year-old patient with known Coat’s disease who developed BRVO 4 days after testing positive for COVID-19 [20]. These cases with significant risk factors for retinal vein occlusions were not excluded from the analysis as a cause–effect relationship cannot be certain but should be dealt with skepticism.

Another possible “sibling pathology” could be any cerebrovascular complication of COVID-19, due to the common embryologic origin with retinal tissue. Patients with cerebrovascular disease and COVID-19 have worse outcomes than those with non-COVID cerebrovascular disease. This may mean that COVID-19 infection worsens outcomes in patients already at high risk for cerebrovascular complications [53,54]. However, the currently available data does not support any probable pathophysiologic mechanism by which SARS-CoV-2 could cause acute ischemic strokes as a primary culprit [55]. Recently, a multicenter study by Li et al., enrolling more than 1 million COVID-19 patients and controls from 46 healthcare organizations in the United States, demonstrated that people suffering from COVID-19 were more prone to develop branch retinal vein occlusions compared to healthy individuals. The authors concluded that COVID-19 may be associated with retinal vein occlusion, strengthening the results of our study [56].

In conclusion, COVID-19 seems to play a role in the pathogenesis of retinal vascular occlusions, as several cases of RVO and RAO have been reported during or after SARS-CoV-2 infections and vaccinations. Several mechanisms can be proposed by examining various clinical entities with vascular complications associated with COVID-19. However, whether SARS-CoV-2 may be strongly associated with an increased risk of retinal vascular events and the possible pathophysiologic mechanisms have not been fully elucidated. Future studies are expected to shed light on this phenomenon.

## Figures and Tables

**Table 1:** Cases of retinal vein occlusion associated with COVID-19 infection or SARS-CoV-2 immunization.

Year, Authors	Age	Sex	Type of Occlusion	Eye	Clinical Symptoms	Time Since COVID-19 Infection or SARS-CoV-2 Immunization (days)	Comorbidities	BCVA at Baseline	Interventions	Final BCVA	Follow-up Period
2020, Gaba et al. [8]	40	Male	CRVO	OU	DV	4 (infection)	Hypertension; Obesity	RE 6/9 LE 6/18	LMWH; Rivaroxaban	RE 6/6 LE 6/12	1 week
2020, Insausti-García et al. [9]	40	Male	CRVO/ Papillophlebitis	OS	DV	42 (infection)	None	20/200	ASA; Bromfenac; Intravitreal dexamethasone implant	20/40	2 weeks
2020, Rego Lorca et al. [10]	30	Female	CRVO	OU	DV, floaters	N/A (infection)	Maturity-onset DM of the young	BE 7/10	N/A	N/A	N/A
2020, Kapatayes et al. [11]	59	Male	CRVO	OD	DV	N/A (infection)	Microscopic colitis	20/20	None	20/20	N/A
2020, Sheth et al. [12]	52	Male	BRVO	OS	DV	10 (infection)	None	6/60	Oral methylprednisolone; Intravitreal anti-VEGF	6/9	1 month
2020, Walinjkar et al. [13]	17	Female	CRVO	OD	DV	22 (infection)	PCOS	6/24	Intravitreal anti-VEGF	6/18	1 month
2020, Yahalomi et al. [14]	33	Male	CRVO	OS	DV, flashes	35 (infection)	None	20/25	None	20/20	Several months
2021, Finn et al. [15]	32	Male	CRVO	OD	VF defect	N/A (infection)	None	20/20	N/A	N/A	N/A
2021, Raval et al. [16]	39	Male	CRVO	OD	DV, floaters	7 (infection)	None	20/150	Intravitreal anti-VEGF	20/30	N/A
2021, Venkatesh et al. [17]	56	Female	CRVO	OS	DV	N/A (infection)	DM	6/18	Low dose ASA	6/6	1 month
2022, Sugihara et al. [7]	38	Male	BRVO	OS	DV	2 (2nd dose of BNT162b2, Comirnaty, Pfizer-BioNTech vaccine)	None	20/25	Intravitreal anti-VEGF	20/20	7 months
2022, Sonawane et al. [18]	50	Male	CRVO	OD	DV	4 (2nd dose of ChAdOx1 nCoV-19, Covishield, AstraZeneca-Oxford vaccine)	DM	6/60	Intravitreal anti-VEGF	N/A	N/A
2022, Sonawane et al. [18]	43	Female	CRVO	OD	DV	3 (2nd dose of ChAdOx1 nCoV-19, Covishield, AstraZeneca-Oxford vaccine)	None	5/60	None	N/A	N/A
2022, Cuadros Sánchez et al. [19]	32	Male	CRVO	OD	DV, photopsia	51 (infection)	None	20/32	Intravitreal dexamethasone implant	20/20	4 months
2022, Garduño Vieyra et al. [20]	43	Male	BRVO	OD	DV	4 (infection)	Coats disease	20/400	Intravitreal anti-VEGF; Periocular triamcinolone; Focal laser treatment	20/20	3 months
2022, Pur et al. [21]	34	Male	BRVO	OD	Inferior VF defect, photopsia	2 (1st dose of BNT162b2, Comirnaty, Pfizer-BioNTech vaccine)	None	20/20	None	20/20	10 months
2022, Tanaka et al. [22]	50	Female	BRVO	OD	DV	3 (1st dose of BNT162b2, Comirnaty, Pfizer-BioNTech vaccine)	Breast cancer (treated with tamoxifen)	20/25	Intravitreal anti-VEGF	20/20	2 months
2022, Tanaka et al. [22]	56	Female	BRVO	OD	DV	3 (1st dose of BNT162b2, Comirnaty, Pfizer-BioNTech vaccine)	None	13/20	Intravitreal anti-VEGF	20/20	2 months
2023, Lin et al. [23]	48	Male	CRVO	OU	DV	14 (infection)	Hypertension; DM type II; CKD stage IV	OU CF	BE Intravitreal anti-VEGF	OU 20/20	Several months
2023, Ishiguro et al. [24]	47	Male	CRVO	OD	DV	8 h (1st dose of BNT162b2, Comirnaty, Pfizer-BioNTech vaccine)	None	20/200	Intravitreal anti-VEGF	20/20	10 months
2023, Lee et al. [25]	41	Female	BRVO	OD	Central VF defect	3 (2nd dose of BNT162b2, Comirnaty, Pfizer-BioNTech vaccine)	None	6/18	Intravitreal anti-VEGF	6/6	1 month

BCVA = Best-corrected visual acuity; CRVO = Central retinal vein occlusion; BRVO = Branch retinal vein occlusion; OD = Right eye; OS = Left eye; OU = Both eyes; DV = Decreased vision; VF = Visual field; CF = Counting fingers; DM = Diabetes mellitus; PCOS = Polycystic ovarian syndrome; CKD = Chronic kidney disease; ASA = Acetylsalicylic acid; LMWH = Low molecular weight heparin; VEGF = Vascular endothelial growth factor; N/A = Not available.

**Table 2:** Cases of retinal artery occlusion associated with COVID-19 infection or SARS-CoV-2 immunization.

Year , Authors	Age	Sex	Type of Occlusion	Eye	Clinical Symptoms	Time Since COVID-19 Infection or SARS-CoV-2 Immunization (days)	Comorbidities	BCVA at Baseline	Interventions	Final BCVA	Follow-up Period
2020, Acharya et al. [6]	60	Male	CRAO	OD	PVL	12 (infection)	Hypertension; Dyslipidemia; Coronary artery disease; COPD	NLP	N/A	NLP	N/A
2020, Montesel et al. [26]	59	Male	CRAO	OS	PVL	21 (infection)	Hypertension; Hyperuricemia; Heterozygous hemoglobin S (sickle cell trait)	LP	None	CF	1 month
2021, Bapaye et al. [27]	42	Male	CRAO	OU	PVL	13 (infection)	None	OU LP	ASA; Oral dexamethasone	OU LP	6 weeks
2021, Murchison et al. [28]	5th decade	Male	CRAO	OD	PVL	2 (infection)	Hypertension	HM	LMWH	HM	N/A
2021, Raj et al. [29]	37	Male	CRAO	OS	PVL, proptosis, ptosis, ophthalmo-plegia	14 (infection)	None	NLP	IV steroids; IV antibiotics; IV anticoagulants; Symptomatic care	NLP	N/A
2021, Sanjay et al. [30]	66	Male	CRAO	OD	PVL	10 (infection)	DM type II	20/2666	Topical prednisolone; Topical anticholinergic	N/A	N/A
2021, Savastano et al. [31]	58	Male	BRAO	OS	None	40 (infection)	Coronary artery disease; Hypertension; Hyperuricemia	55/55	None	55/55	1 week
2021, Turedi et al. [32]	54	Male	CRAO	OD	PVL	14 (infection)	None	CF	Anti-glaucoma eye drops; Hyperbaric oxygen therapy; Ocular massage	CF	5 days
2022, Abdin et al. [33]	76	Female	CRAO	OS	PVL	2 (1st dose of ChAdOx1-S [recombinant], Vaxzevria, AstraZeneca-Oxford vaccine)	Hypothyroidism	HM	ASA; IV vasodilator; Anti-glaucoma eye drops; Ocular massage	N/A	N/A
2022, Chow et al. [34]	70	Male	CRAO	OD	PVL	5 (1st dose of mRNA-1273, Spikevax, Moderna vaccine)	Hypertension; Dyslipidemia	CF	Clopidogrel; Hyperbaric oxygen therapy	CF	4 months
2022, Thakar et al. [35]	44	Male	CRAO	OS	PVL	10 (1st dose of BBV152, Covaxin, Bharat Biotech vaccine)	None	LP	None	N/A	N/A
2023, Rv et al. [36]	68	Female	CRAO	OS	PVL	N/A (infection)	Hypertension	20/400	Anti-glaucoma eye drops; Ocular massage;	CF	4 months
2023, Heidarzadeh et al. [37]	44	Male	CRAO	OS	PVL	20 (infection)	None	LP	Oral prednisolone; Anti-glaucoma eye drops; Panretinal photocoagulation	NLP	N/A
2024, Kunihiko et al. [38]	43	Female	BRAO	OD	DV	33 (infection)	None	20/25	IV vasodilator	N/A	6 months
2024, Jiang et al. [39]	76	Male	BRAO	OS	DV	12 (infection)	Hypertension	6/20	ASA; LMWH; Oral prednisolone; IV vasodilator; Retrobulbar anticholinergic; Anterior chamber puncture; Supplemental oxygen	20/20	12 months

BCVA = Best-corrected visual acuity; CRAO = Central retinal artery occlusion; BRAO = Branch retinal artery occlusion; OD = Right eye; OS = Left eye; OU = Both eyes; DV = Decreased vision; PVL = Painless vision loss; DM = Diabetes mellitus; COPD = Chronic obstructive pulmonary disease; NLP = No light perception; LP = Light perception; HM = Hand motion; CF = Counting fingers; ASA = Acetylsalicylic acid; LMWH = Low molecular weight heparin; IV = Intravenous; N/A = Not available.

## Data Availability

The raw data supporting this study’s findings are available from the corresponding author upon request.
